# Perspective: Should Exclusive Breastfeeding Still Be Recommended for 6 Months?

**DOI:** 10.1093/advances/nmz039

**Published:** 2019-05-31

**Authors:** Rafael Pérez-Escamilla, Gabriela S Buccini, Sofia Segura-Pérez, Ellen Piwoz

**Affiliations:** 1 Yale School of Public Health, New Haven, CT; 2 Hispanic Health Council, Hartford, CT; 3 Bill & Melinda Gates Foundation, Seattle, WA

**Keywords:** exclusive breastfeeding, complementary feeding, food allergies, iron deficiency, anemia, breast milk, policy

## Abstract

The WHO recommends exclusive breastfeeding of infants for the first 6 mo of life (EBF-6). We reviewed the evidence behind concerns related to this recommendation. The risk of iron deficiency among EBF-6 infants can be significantly reduced if delayed cord clamping is performed in all newborns. At the moment there is no population-level evidence indicating that exclusive breastfeeding for 6 mo compared with <6 mo increases the risk of developing food allergies. Mild to moderate maternal undernutrition may reduce amounts of some nutrients in breast milk but does not directly diminish milk volume. Persistent reports of insufficient milk by women globally are likely to be the result of lack of access to timely lactation counseling and social support rather than primary biological reasons. All newborns should have their growth, hydration status, and development carefully monitored. In instances where formula supplementation is required, it should be done under the guidance of a qualified provider taking into account that early introduction of breast-milk supplements is a risk factor for early termination of exclusive breastfeeding and any breastfeeding. We found no evidence to support changes to the EBF-6 public health recommendation, although variability in inter-infant developmental readiness is recognized. We suggest that infant and young feeding guidelines make clear that complementary foods should be introduced at around 6 mo of age, taking infant developmental readiness into account.

## Introduction

In 2001, the WHO recommended exclusive breastfeeding (EBF) for the first 6 mo of life (EBF-6), replacing its previous recommendation of EBF for 4-6 mo (1, 2). EBF-6 was reaffirmed in 2012 (3); however, concerns continue to be raised about the feasibility and safety of this recommendation, and some have called for its reexamination (4). The objectives of this Perspective are to: *1*) present and discuss the evidence on which the WHO EBF-6 recommendation is based; *2*) review evidence behind concerns related to EBF-6 including iron-deficiency anemia, food allergies, and maternal malnutrition; *3*) clarify EBF-6 in the context of a public health recommendation compared with an individual-level recommendation; and *4*) identify knowledge gaps. The literature reviewed was identified through a scan of review articles, recommendations from professional societies, scientific reports from the WHO, and the National Academies of Science, Engineering and Medicine; the authors’ files; and by eliciting feedback from experts based in academic institutions and at UNICEF, the WHO, and the Bill & Melinda Gates Foundation.

## Evidence behind the EBF-6 Recommendation

The 2001 WHO EBF-6 recommendation was based on evidence gathered from a systematic review comparing EBF for 6 mo to EBF for 3-4 mo with continued BF for at least 6 mo, examining the impact of these practices on child health, growth, and development, as well as on maternal health (1, 2). Evidence was gathered from 20 studies in low-income/middle-income countries (LMICs) (*n* = 9) and high-income countries (*n* = 11). All but 2 of these studies were observational. The 2 randomized controlled trials (RCTs) were conducted in Honduras and included low-birth-weight (LBW) and adequate-birth-weight term (≥37 wk) infants who were receiving EBF for different durations. In both studies infants were provided with safe and nutritious complementary foods (5, 6). The first of these RCTs found that *1*) there was no weight gain or linear-growth benefit for introduction of complementary foods at age 4 mo instead of age 6 mo (7); and *2*) EBF-6 (compared with EBF-4) increased the risk of iron deficiency as indicated by low hematocrit, especially among LBW infants (8). The second trial, conducted among LBW infants only, also found no growth advantage during the first year of life for EBF-4 compared with EBF-6 (6). Data from both trials indicated that EBF-6 infants crawled sooner and in the first trial they also walked earlier compared with EBF-4 infants (9). Based on the results from RCTs and observational studies included in this review, it was concluded that: *1*) no significant differences were found in growth among EBF-6 children compared with those with other EBF durations; *2*) a lower incidence of gastrointestinal infection among EBF-6 infants was found when compared to infants receiving EBF for shorter durations; and *3*) there was a potential risk for development of iron-deficiency anemia before age 6 mo, especially among exclusively breastfed infants born with suboptimal iron reserves and among LBW infants (1). In 2012, the WHO commissioned an updated systematic review of the evidence on EBF duration. This updated review included 23 studies, 3 of which were not included in the first review, 11 from LMICs, and 12 from high-income countries (3). This more-recent systematic review, which followed a full guideline review committee process (10), reaffirmed the WHO 2001 EBF-6 recommendation (1). Similar to the findings from the Honduran studies, an RCT conducted in Iceland found no infant growth advantage for EBF-4 compared with EBF-6 infants. Length and head circumference were measured at birth, 6 wk, and 3, 4, 5, 6, 8, 10, and 12 mo of age; and weight and height were measured at 18, 29, and 38 mo (11). An observational analysis of the Promoting Breastfeeding Interventional Trial in Belarus, a country with an adequate sanitation infrastructure, reported that EBF-6 infants had comparable growth and lower risk of gastrointestinal infections when compared to EBF-3 infants, consistent with evidence from low and LMIC settings (12).

Because RCTs are the gold standard for making causal inferences, the scarcity of RCTs underlying the WHO EBF-6 recommendation is seen by some as a weakness. However, the numerous maternal and child health benefits associated with breastfeeding (13–19) make experimental research designs impractical and unlikely to receive ethical approval in many settings.

## Concerns about the EBF-6 Recommendation

### Iron-deficiency anemia

Iron-deficiency anemia during early infancy has been identified as a risk associated with EBF-6, with potential long-term motor and mental developmental consequences (20). Although iron in human milk is highly bioavailable, breast milk contains relatively small amounts of iron that may not be able to sustain adequate iron status until age 6 mo, particularly among infants who have inadequate iron endowments at birth either because they are born to a mother with iron deficiency or they are preterm or LBW (21–23).

Addressing potential iron deficiency in infants is a challenge because supplementing the mother's diet with iron does not increase iron concentrations in her breast milk (24, 25) and giving iron drops to infants before age 6 mo has been ineffective in settings with weak healthcare systems (26, 27). This has led some to argue that the risk of iron deficiency through EBF-6 implementation outweighs this recommendation's other benefits, and that iron-rich complementary foods should be introduced to infants as early as age 4 mo (8, 11, 28, 29). This risk-benefit assessment does not, however, take into consideration the risk of infant morbidity and mortality associated with unsafe, home-prepared, complementary foods often found in low-income settings (30–32).

**FIGURE 1:**
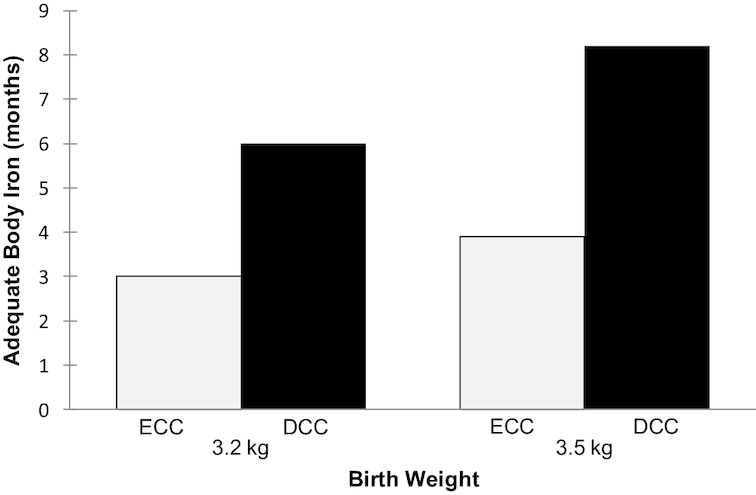
Duration of adequate body iron after birth by birth weight and timing of umbilical-cord clamping. Developed by authors from evidence reported by Chaparro and Lutter (22). ECC, early cord clamping; DCC, delayed umbilical-cord clamping.

The risk for iron deficiency in the first 6 mo of life can be successfully mitigated by delayed umbilical-cord clamping (22, 33, 34) (**Figure 1**). The amount of iron provided from stores at birth plus intake from breast milk can provide sufficient iron for 6 mo if the exclusively breastfed infant is born at term, normal birth weight, the mother had adequate prenatal iron status, and the infant underwent delayed cord clamping. In contrast, early cord clamping in the same infant would provide sufficient iron for 3 mo only (34). LBW infants also benefit from delayed cord clamping. However, the WHO recommends medicinal iron drops beginning at age 2–3 mo for LBW infants, negating the need for early complementary feeding to reduce iron-deficiency risk (35).

The delayed cord clamping recommendation was issued by WHO based on strong evidence, and was subsequently incorporated into the WHO's Essential Newborn Care Course in 2012 (36) and to an official WHO Guideline in 2014 (37).

From the available evidence and WHO recommendations, we conclude that there is no need to change the EBF-6 recommendation based on concerns about iron-deficiency anemia. This condition can be addressed through delayed cord clamping and medicinal iron and does not require infant food introduction before 6 mo.

### Food allergies

A food allergy is an adverse immune response to a component of a specific food (38). The majority of food allergies are caused by 8 foods: eggs, milk, peanuts, tree nuts, fish, shellfish, wheat, and soy (38). Between 1997 and 2007 in the United States, the prevalence of food allergies among children aged <18 y increased by 18% (39). In 2016, 6.5% of children in the United States suffered food allergies (40). A population-based parent survey conducted in the United States at around the same time reported a slightly higher prevalence of food allergies among children aged <18 y (7.6%), with peanut allergy being the most commonly reported (41). The HealthNuts cohort study in Australia reported a prevalence of food challenged-confirmed allergies of 11% at age 1 y and of 3.8% at age 4 y (42). The reported prevalence of food allergies varies considerably among countries. For example, a review of studies with adequate diagnosis of food allergies among children aged <6 y reported wide ranges in the following countries: 1.1% to 7.7% in the upper-middle-income countries of Thailand, South Africa, and China (43–45); 1.9% to 10.4% in the high-income countries of Iceland and Denmark (46, 47), the Isle of Wight in England (48), the United Kingdom (49), Norway (50), and Australia (51).

Because food allergies can lead to serious health problems, including anaphylactic reactions and premature death, there is an interest in better understanding of whether, and how, they are related to diverse infant-feeding practices (52, 53). Thirteen reviews and an expert report from the National Academies of Sciences, Engineering and Medicine (52, 53) found a lack of evidence to support the previous recommendations to delay introduction of potentially allergenic foods (specifically peanuts, eggs, fish, and gluten-containing foods) beyond age 12 mo to reduce the risk of allergies. Evidence from high-income countries has led to an interim clinical recommendation indicating that to prevent food allergies, infants at risk of developing them should be exposed to potentially allergenic foods between the ages of 4 and 6 mo (53, 54). This clinical guidance, endorsed by 10 allergy societies/academies from Australia, the United States, Canada, Europe, Japan, and the Middle East, states that such early exposure should be implemented on an individual basis, under the supervision of a qualified healthcare professional. The strongest evidence for this recommendation pertains to peanut and egg allergies based on findings from a single RCT conducted in the United Kingdom (55). The United Kingdom's Learning Early About A Peanut Allergy trial found that exposing infants from age 4 to 11 mo, who were at high risk of peanut allergy, to small tastes of peanut butter reduced the risk of developing allergies to these foods later in life (55). This trial used the term “high risk” for infants with severe eczema or who tested positive for an egg allergy. Interestingly, although it was initially reported that the protective association of peanut exposure was stronger the earlier the introduction took place (specifically, between the ages of 4 and 6 mo) (56), a subsequent analysis showed that protection was significantly higher with peanut introduction between 6 and 11 mo, compared to the challenge between 4 and 6 mo (57). The United Kingdom's Enquiring about Tolerance (EAT) trial focused on EBF infants randomly assigned to introduction of 6 potentially allergenic foods at age 3 mo (compared with age 6 mo): cooked egg, peanut, cow milk, sesame, white fish, and wheat (58). EAT found that the exposed infants were less likely to develop food allergies compared with their counterparts who continued EBF for about 6 mo (59). This protective effect may be the result of “training” the immune system to recognize these foods during a sensitive time in development of the infant's immune system (52). A study limitation of EAT was low compliance with the intervention protocol. However, analysis of the per-protocol group showed that the food-allergy incidence was significantly lower in the intervention group (2.4%) compared with the control group (7.3%, *P* = 0.01) (58). The EAT intervention most strongly protected against allergy to peanuts (with a dosage similar to that used in the Learning Early About A Peanut Allergy trial) and eggs.

Recommendations on the introduction of potentially allergenic foods in high-income countries remains controversial. The United Kingdom's Scientific Advisory Committee on Nutrition recently concluded (60) that whereas “the available evidence indicates that the deliberate exclusion or delayed introduction of peanut or hen's egg beyond 6 to 12 months of age may increase the risk of allergy to the same foods, the available evidence indicates that allergenic foods such as peanut, hen's egg, gluten, or fish can be introduced from around 6 months of age.” Furthermore, the Scientific Advisory Committee on Nutrition specifically concluded that “there is insufficient evidence to demonstrate that the introduction of peanut or hen's egg into the infant diet before 6 months of age reduces the risk of developing food allergy to any greater extent than introduction from around 6 months of age.”

A recent RCT from Ecuador found that introducing eggs between 6 and 9 mo and feeding 1 daily for 6 mo improved linear growth without increasing the risk of egg allergy (61). Furthermore, a systematic review that included 5 RCTs found that introduction of eggs between ages 4 and 6 mo was associated with a reduction in risk for egg allergies, independent of the level of risk that the infants had for developing them (62).

A previous evidence review concluded that changes to the EBF-6 public health recommendation were not needed because of general concerns about severe food allergy, but individual infants suspected to be at high risk should be assessed by a qualified provider to determine potential risk of developing severe allergy to certain foods and timing for their introduction (52). Recent studies suggest that breastfeeding may protect against development of food allergies and that this relation may be influenced by maternal diet. The Canadian Asthma Primary Prevention Birth Cohort study examined the relation between maternal peanut consumption while breastfeeding, timing of peanut introduction to the child diet, and peanut sensitization at age 7 y. This study found the lowest incidence of peanut sensitization (allergy risk) among mothers who ate peanuts while breastfeeding and introduced peanuts to their child before age 12 mo (63). This finding may have public health implications as a meta-analysis of 15 birth cohorts—mostly located in Europe—found that food sensitization during infancy, assessed by skin prick testing or measuring serum IgE during the first 2 y of life, was associated with eczema in late infancy, wheeze/asthma, and allergies during childhood (64).

Based on findings from the Canadian Healthy Infant Longitudinal Development cohort study, Miliku and Azad (65) concluded that the human milk oligosaccharide (HMO) composition of human milk is associated with development of food sensitization in the first year of life. HMO composition varies between maternal-infant dyads, and may be influenced by maternal diet and other environmental exposures, as well as lactation stage, degree of breastfeeding exclusivity, and genetics (e.g. fucosyltransferase-2 secretor status); and is a strong determinant of the microbiome. Findings on the relation between maternal diet and HMO composition are inconsistent (66), however, and further research is warranted to better understand these relations.

In summary, providing small tastes of potentially allergenic foods under medical supervision to 4 to 6 mo old infants at high risk of developing food allergies (such as infants with a family history of allergies to eggs and peanuts) has been endorsed by several professional allergy societies in high-income countries for the purpose of preventing future food allergies (52–54, 67). However, more recent expert reviews do not support this recommendation (60). Hence, this issue requires further high-quality evidence from diverse settings. Recent studies suggest that maternal diet may modify the impact of early exposure to potentially allergenic foods and subsequent risk of food allergies in the breastfed infant, but this finding also requires further confirmation and better understanding of underlying biological mechanisms.

It is important to note that the aforementioned studies were carried out in populations of infants at risk of developing severe food allergy. There is presently no population-level evidence indicating that EBF for 6 mo compared with <6 mo increases the risk of developing food allergies. Hence, a change to the EBF-6 recommendation for the population at large to prevent food allergies is unwarranted.

### Self-reported insufficient milk

**FIGURE 2:**
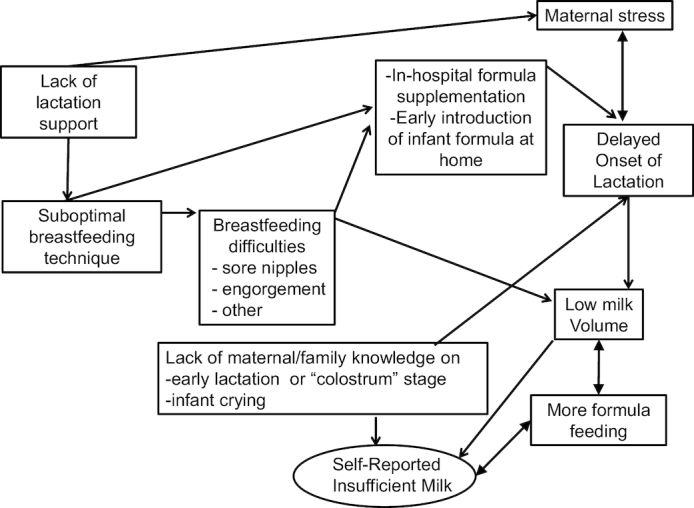
Hypothetical model proposing how lack of access to lactation support can lead to SRIM. Original model prepared by authors from evidence reported by Dewey et al. (96), Segura-Millán et al. (70), Kent (105), Kent et al. (72, 73), Wood et al. (94), Pérez-Escamilla et al. (74), Chapman and Pérez-Escamilla (80), and Giugliani (81).

Self-reported insufficient milk (SRIM) is the most commonly mentioned reason provided by women the world over for introducing breast-milk substitutes into the infant's diet or for stopping breastfeeding altogether (68–70). Some have hypothesized that SRIM is simply a socially accepted excuse that women give for explaining why they are not practicing what they know is recommended infant-feeding behavior (71). However, others have postulated that SRIM may result from not understanding the lactation process, as women often report SRIM within the first 2 d after birth, a time when only small amounts of colostrum are being produced, and they introduce breast-milk substitutes in response to this (i.e., pre-lacteal feeding) (72–74). SRIM may also be the result of perceptions from healthcare providers about the capacity of women to produce enough milk to satiate their infants (73, 75). The precise proportion of women who cannot produce enough milk for satiating and meeting the nutritional needs of their infants for primary biological reasons remains unknown (76). However, it is likely that this proportion is low because the lactation process is mainly driven by a highly protected infant demand-maternal supply process (77, 78). As the evidence presented below indicates, lack of timely lactation management and social support appear to be the strongest predictors of SRIM and other lactation difficulties (70, 72, 79–81) (**Figure 2**).

The evidence suggests that delayed onset of lactation (DOL), i.e., when the copious production of milk does not begin within 72 h postpartum, increases the risk of SRIM and short breastfeeding durations, and can result from pre-lacteal feeds (i.e., foods and fluids other than breast milk given before the onset of lactation or “milk arrival”) that interfere with the infant's suckling process (74). Modifiable risk factors for DOL include maternal stress, maternal obesity, and in-hospital formula supplementation (82–85). Multiparous Guatemalan women with higher salivary amounts of cortisol (a stress hormone) during labor were more likely to have DOL (83). Chen et al. (82) found an association among stress indicators including longer labor duration, maternal exhaustion, increased stress hormones, and DOL. Consistent with these findings, Chapman and Pérez-Escamilla (80) found in a sample of American women that unscheduled cesarean delivery or vaginal delivery with a prolonged second stage (the “pushing” stage) of labor were risk factors for DOL. Living under poverty conditions does not seem to increase the risk of DOL. In fact, DOL appears to happen less frequently in low-income countries, especially in settings where the human birth process is less medicalized (80, 86). By contrast, maternal obesity has been consistently identified as a risk factor for DOL in low-, middle-, and high-income countries (80, 85). Likewise, prenatal insulin profiles denoting poor blood glucose control have been associated with DOL in the United States (87). The importance of early initiation of breastfeeding as a means for reducing interference with normal lactation processes is suggested in data from 51 countries, which indicate that timing of breastfeeding initiation is a significant risk factor for early introduction of supplementary (pre-lacteal) feeds (88).

In summary, SRIM leads to shortened breastfeeding durations, perhaps as a result of introduction of pre-lacteal feeds in response to DOL or perceptions of breastfeeding inadequacy as a result of infant crying, not feeling breasts full, or not seeing milk flowing from nipple (70, 89, 90). Studies have consistently shown that providing infant formula to neonates before the mother's milk comes in—which can be in response to SRIM—increases the risk for interference with the demand-supply process and hence can shorten breastfeeding duration (74, 91, 92). The evidence suggests that it is likely most cases of SRIM are the result of poor lactation management and social support and could be prevented if addressed antenatally and within the first 2 wk postpartum with skilled counseling and support (70, 72, 93–96). Therefore, for the population at large, maternal concerns about milk supply do not justify revision of the EBF-6 recommendation. This condition does, however, necessitate provision of timely breastfeeding support, particularly as lactation is established. The WHO and UNICEF (97) recommendation to initiate breastfeeding within the first hour of life, and to offer the breast exclusively and on demand to facilitate the establishment of a milk supply, can help to mitigate this problem.

### Maternal nutritional status and breast milk quality

The WHO committee issuing the EBF-6 recommendation in 2001 acknowledged the dearth of evidence they had available to assess how EBF impacts maternal nutrition. It also recognized the lack of evidence on human milk meeting all infant nutrient requirements (2). Furthermore, the committee did not have the evidence available to assess the nutrient adequacy of human milk output in the first 6 mo across different populations (98). In fact, to this day, reference standards for human milk volume and composition do not exist, although these are currently being explored (77, 99, 100).

Recognizing these limitations, the WHO guideline development group (98) concluded that: *1*) infant protein and energy needs can be met through EBF-6; *2*) the adequacy of some nutrients (such as vitamin A and vitamin B-6) depend on maternal diet; *3*) calcium quantity in breast milk is tightly regulated and does not depend on maternal intake; *4*) human milk is low in iron and by 6 mo is also relatively low in zinc, and the concentrations of these nutrients in breast milk cannot be altered through maternal supplementation; *5*) the potential for the EBF-6 infant to become iron-deficient should be considered, especially among LBW/premature infants. These conclusions, which echoed the Institute of Medicine (IOM) 1991 seminal report on human lactation (101), have been reaffirmed over time (25, 102).

Despite decades of research on human lactation, we still lack a fundamental understanding of what is the normal range of breast-milk production and human milk nutrient composition. This is in large part because there are still many uncertainties about infant nutrient requirements during the first 6 mo of life and hence there is a lack of blood thresholds for identifying micronutrient deficiencies (99, 100, 103). Understanding the physiology of lactation sheds light on some of these outstanding questions. Therefore, in the following sections we examine drivers of milk synthesis and nutrient transport.

#### Human milk synthesis

Lactocytes or milk-producing cells lining the inner walls of the mammary gland alveoli become activated for milk synthesis in response to infant suckling (104). Human milk synthesis involves complex and highly coordinated mechanisms in transport of maternal-blood nutrients into the alveolar lumen (105). The process of milk synthesis takes place via exocytosis, secretion of lipid droplets, transcellular transport through the lactocyte, or paracellular transport through lactocyte junctions. The synthesis of human milk proteins, fat, and lactose involves different lactocyte organelles before secretion into the alveolar lumen (106). The mammary gland can tightly control the concentration of minerals, such as calcium, iron, zinc, and copper, to reduce the risk of nutrient deficiencies or excesses in the infant (107, 108). This process involves numerous protein transporters and steps highly synchronized with each other. Transport of calcium, electrolytes, and water-soluble vitamins occurs through transcellular pathways involving complex protein transport systems working in concert with each other (108). Human milk synthesis is an energy- and nutrient-intense process. Once lactation has been established, mothers produce a mean of 750 g/d breast milk, although there is wide variation among women (from 440 g/d to 1220 g/d) (103), in part driven by infant demand. On average, women need an additional 500 kcal/d to support lactation, compared to 250 kcal/d during the second trimester of pregnancy and 452 kcal/d during the third trimester (103).

#### Maternal nutrition status and human milk composition and volume

Mild to moderate maternal undernutrition can lead to suboptimal amounts of some key micronutrients in human milk (98). The WHO EBF-6 recommendation could not take human milk nutritional quality fully into account because of a lack of data on breast-milk composition in different settings. Although the evidence is relatively scant, available data show significant variation across populations in both human milk composition and infant response to maternal nutrient supplements (98). Some evidence shows that for some of the nutrients that can be increased in breast milk through maternal supplementation (i.e., group 1 nutrients), very high supplementation would be needed to achieve modest nutrient increases in the infant's status for the corresponding nutrient (see supplementation section below).

Early infant supplementation may be advised on an individualized basis, when 1 or more nutrient deficiencies are detected. Indeed, infant vitamin and mineral supplementation is permitted within the WHO definition of EBF.

Regarding macronutrient composition, evidence suggests uniformity in mean human milk protein, fat, carbohydrate (lactose), and energy concentration across populations, regardless of maternal nutritional status (101, 102). This is particularly true for protein and lactose (109–118) (**Table 1**).

**TABLE 1:** Macronutrient concentrations of mature human milk^1^

Term infants mature milk	Country	Study reference	Protein, g/dL	Fat, g/dL	Lactose, g/dL	Energy, kcal/dL
	High-income countries^2^	Gidrewicz and Fenton (109)	0.9 (0.6–1.2)	3.4 (1.6–5.2)	6.8 ± 0.3	68 (50–86)
Fore milk, hind milk^3^	Finland	Saarela et al. (110)	1.1 ± 0.1 (0.1–1.1)	1.9 ± 0.4, 5.7 ± 2.4	7.6 ± 0.5, 7.1 ± 0.2	53.1 ± 4.3, 86.3 ± 21.0
	United States	Nommsen et al. (111)	1.2 (0.9–1.5)	3.6 (2.2–5.0)	7.4 (7.2–7.7)	70 (57–83)
	Brazil	Gomes et al. (112)	1.1 (1.1–1.3)	2.8 (2.0–3.8)	7.1 (6.7–7.3)	-
	Bangladesh	Brown et al. (113)	-	2.8 ± 0.60	7.9 ± 0.4	61.0 ± 6
	Israel, <35-y-old women	Lubetzky et al. (114)	0.9 ± 0.4 (0.2–1.9)	4.6 ± 1.1 (2.6–7.0)	5.2 ± 0.7 (4.0–6.7)	71.5 ± 12.7 (47–100)
	Israel, ≥35-y-old women	Lubetzky et al. (114)	0.9 ± 0.3 (0.5–1.7)	4.1 ± 1.1 (1.3–6.0)	5.8 ± 0.9 (4.3–6.9)	67.9 ± 19.4 (36–90)
	Korea	Chang et al. (115)	1.4 ± 0.3 (0.5–2.9)	3.0 ± 1.4 (0.2–9.5)	7.1 ± 0.4 (4.2–9.9)	61.1 ± 13.1 (43.2–104.2)
Donor samples		Wojcik et al. (116)	1.2 (0.7–1.7)	3.2 (1.2–5.2)	7.8 (6.0–9.6)	65 (43–87)
		Michaelsen et al. (117)	0.9 (0.6–1.4)	3.6 (1.8–8.9)	7.2 (6.4–7.6)	67 (50–115)
		Saarela et al. (110)	1.1 ± 0.1	3.2 ± 1.1	7.3 ± 0.4	64.7 ± 10.8
Reference standard		American Academy of Pediatrics (118)	0.9	3.5	6.7	(65–70)

^1^Values are means ± SDs and/or (range).

^2^Australia, Canada, France, Finland, Germany, Japan, Italy, the Netherlands, Spain, Sweden, and the United States.

^3^Fore milk refers to breast milk sampled at beginning of the nursing episode; hind milk refers to milk sampled toward the end of the nursing episode.

This finding is consistent across studies even when different human milk sampling and analysis methods are used to determine macronutrient content; and also, when socioeconomic, nutritional status, and demographic characteristics—including infant age at which milk samples were obtained—vary within the populations assessed.

In the field of human milk composition, vitamins and minerals are classified into 2 groups, depending on whether or not they are responsive to maternal nutritional status (25) (**Table 2**). Group 1 nutrient deficiencies can be addressed through public-health measures as their concentrations in human milk are negatively affected by maternal depletion and their concentration in breast milk can potentially be increased through changes in maternal diet or via supplementation (98, 103). By contrast, group 2 nutrient amounts in breast milk are unrelated to maternal nutritional status, as seen in studies of undernourished and well-nourished populations in Honduras and Sweden (119). Although long-chain PUFAs, which are crucial for the child's brain development have not been previously listed as group 1 nutrients, it is important that they are recognized as such as their amounts in breast milk are responsive to maternal diet and supplementation (102). Expert panels suggest that adult women ingest ω-3 fatty acid-rich fish sources and/or supplements to consume a minimum of 300–1000 mg DHA/d while pregnant or breastfeeding (120–122).

**TABLE 2:** Group 1 and Group 2 nutrients in human milk^1^

Nutrient type	
Group 1	Group 2
Thiamin	Calcium
Riboflavin	Folate
Vitamin B-6	Iron
Vitamin B-12	Copper
Choline	Zinc
Retinol	Vitamin K
Vitamin A	
Vitamin D	
Selenium	
Iodine	
PUFAs	

^1^Group 1 nutrients can be increased in breast milk through maternal supplementation, but group 2 nutrients cannot. Based on evidence reported by Allen (25) and Valentine and Wagner (102).

##### Effects of maternal supplementation on human milk quality

When a breastfeeding mother ingests nutrient supplements, complex metabolic processes may allow for only a very small amount of these supplement-derived nutrients to enter the breast milk. Indeed, wide variability exists in how individual infants respond to similar amounts of maternal supplementation, and for some nutrients the amounts of maternal supplementation would need to be exceedingly high to meet infant requirements (123, 124). This variability in response to micronutrient supplementation is a function of the specific micronutrient, amount of supplementation, and maternal nutritional status. Differential responses to various micronutrient supplements are described in the paragraphs that follow.

A 2013 review confirms the presence in breast milk of nutrients that are responsive (group 1) and nonresponsive (group 2) to maternal nutritional status in low- and high-income country settings (123). For group 1 nutrients, earlier studies among thiamine-deficient women in Gambia and India (125, 126) demonstrated strong human milk response to maternal thiamine supplementation. In the Gambia study, both maternal and infant thiamine status improved in <2 wk after supplementation. These 2 studies showed that human milk responds to maternal riboflavin supplementation, and that riboflavin status rapidly improved in both mothers and infants after maternal supplementation was started. Another study in Guatemala showed a relatively small human milk response to vitamin B-12 supplementation among women deficient in this vitamin (127).

With regard to group 2 nutrients, studies in a rural Mexican Otomi population confirmed that maternal folate status is unrelated to folate concentration in human milk, and that folate concentration in breast milk cannot be altered through maternal supplementation (128). EBF infants generally have adequate folate status, however, irrespective of maternal folate concentrations (25). Therefore, folate is a good example of a nutrient that gets prioritized for the mammary-gland usage, for transfer to the infant, if needs be at the expense of maternal folate status.

Among urban Bangladeshi women, the effects of maternal supplementation on human milk content were detected within 2–4 h for thiamin, riboflavin, vitamin A, and vitamin B-6, although the fraction transferred to breast milk ranged from 0.1% to 6.17% (129). Response to vitamin A supplementation varies across studies, perhaps as a function of maternal vitamin A deficiency. Although studies have consistently demonstrated that maternal vitamin A supplementation impacts its presence in breast milk (101, 102), a recent study conducted in the eastern region of Ghana found that daily supplementation with small-quantity lipid-based nutrient supplements covering the recommended daily intake of vitamin A did not increase breast-milk retinol concentrations (130). The authors attribute this finding to the strong likelihood that women already had adequate vitamin A status.

Concentrations of vitamin D in human milk correlate with maternal vitamin D intake and sunlight exposure. The concentration of vitamin D in breast milk, even for healthy lactating women, is low. Breast milk does not provide infants with adequate vitamin D intake, even in populations typically known to be well nourished, and especially when infants are not exposed to sufficient sunlight (124). Because vitamin D synthesis is stimulated by sunlight on the skin and not obtained in significant amounts from the diet, breastfed and formula-fed infants without sufficient exposure and synthesis may require supplementary vitamin D. Studies in Finland and the United States show that very high maternal vitamin D supplementation is needed (≥2000 IU/d) to provide a significant amount of breast-milk vitamin D for the infant (123, 124). In fact, some studies suggest that as much as 6400 IU/d would be needed to bring the infant's vitamin D status to the desired range while simultaneously protecting the vitamin D status of the mother. This amount of supplementation is so high (about 10 times the daily recommended intake for vitamin D) that this explains in part why the public-health measure currently endorsed for improving vitamin D status of all breastfed infants is based on directly supplementing the infant with this vitamin (400 IU/d) (131).

##### Infant self-regulation of breast-milk intake

Nutrient composition of human milk is a contributing factor to the nutritional status of the EBF infant. However, it is not the only contributor, as infants can self-regulate energy and nutrient intakes by adjusting the volume of milk they consume. The Honduras RCT mentioned previously found that all groups of infants exclusively breastfeeding for 6 mo compared with 4 mo (5) consumed the same mean daily energy intake, indicating that they adjusted their milk-volume intakes in spite of variation in breast-milk fat and energy content. Indeed, an observational analysis of the Honduras trial data documented that infants whose mothers produced higher-energy milk consumed less milk volume compared with infants consuming milk with lower-energy densities (132). By extension, the self-regulation in energy intake also impacts total consumption of other nutrients.

The finding that infant demand drives breast-milk production and subsequent consumption has been reported in multiple studies in high-income countries (133, 134). For example, in a study conducted in the United States among exclusively breastfed infants whose mothers increased their milk supply by expressing additional breast milk for 2 wk, breast-milk intake was driven by infant demand and not by increased maternal breast -ilk production capacity (134). Another study conducted by the same research group in the same population found that residual breast-milk volume, defined as total volume extracted minus mean milk intake in a 24-h period to predict milk volume left in the breast when the infant had completed nursing, was around 100 g/d, and this applied to infants consuming relatively low amounts of breast milk (<650 g/d) (135). A study conducted in the United Kingdom (136) that allowed for mothers to randomly select the breast from which to feed the baby first, found that intake from the second breast was only about 60% of the amount consumed from the first breast.

Stuff and Nichols (137), demonstrated in the United States that energy intake per kg of body weight did not increase after solids were introduced into the diet of exclusively breastfed infants. Nommsen et al. (111) found among 6-mo-old infants from the United States breastfed on demand that solid foods displaced energy intake from breast milk. These findings provide strong evidence that infants have a strong capacity to self-regulate breast-milk intake.

In summary, despite evidence documenting that human milk responds to maternal nutritional status for some key nutrients for which the infants in low-income countries may be compromised, we have gaps in our knowledge about how several nutrients are metabolized and transported in human milk, and this has hindered the development of specific public health recommendations for nursing mothers (99). Questions exist with regard to the nutrient amounts in human milk that provide infants with optimal health and development, while at the same time protecting maternal health and wellbeing (98). The relation between maternal nutritional status and human milk nutrient composition is not straightforward, even for group 1 nutrients (98). Nutrients absorbed by the mother's gastrointestinal tract enter her bloodstream, travel to the breast lactocytes, and cross the lactocytes’ membranes to enter her milk. The suckling infant ingests these nutrients, which are absorbed by the infant's gastrointestinal tract into the bloodstream, and then enter the infant's body tissues. The nutrient concentrations circulating in the infant's blood are therefore a function of the volume of breast milk consumed, the microbiota, and other factors including infections or inflammation (138). Infants have the capacity to self-regulate breast-milk intake as a function of milk energy density, therefore it is difficult to predict nutrient deficiency risk solely based on human milk composition. Hence, currently we do not see a basis for changing the EBF-6 public health recommendation based on concerns about suboptimal maternal dietary intakes.

## Infant Developmental Readiness for Complementary Foods

Increasingly, infant-feeding guidelines, especially those from high-income countries, are acknowledging that there is a relatively wide variability on the exact age at which individual infants are ready to start complementary feeding (139–142). From a child-development perspective, this individual-level variability in readiness to start consuming solid foods should be expected, given that in all developmental domains that have been studied—including crawling, walking, and language—children achieve the same developmental milestones at quite different ages, even when living in relatively similar environments (143–145).

Evidence indicates that most infants will be developmentally ready to handle and benefit from the introduction of complementary foods at around age 6 mo (139, 142, 146, 147). However, some infants may be ready earlier or later (146–150). The key signals that caregivers should pay attention to are the infant's ability to sit with little or no help; to munch/chew and swallow soft, solid foods; to have lost the extrusion reflex (the projection of food from the mouth); and to demonstrate interest in food (151). Achieving these developmental milestones strongly correlates with the maturation of the gastrointestinal tract, kidneys, and immune system required to benefit from introducing complementary foods (147, 148).

## Discussion

The WHO EBF-6 recommendation issued in 2001 (1, 2) was reaffirmed in 2012 (3), and based on our analysis of the literature today, there is still no basis for changing it. Valid concerns have been expressed regarding how this recommendation may not be optimal for certain subgroups of infants, e.g., those born with low iron stores, at high risk of developing food allergies, or not being able to consume an adequate volume of milk. However, in each instance there are interventions that can be tailored to the needs of specific infants rather than changing the EBF-6 recommendation for the population of infants at large. As shown in this Perspective, the risk of development of iron-deficiency anemia can be greatly reduced, even among those at highest risk (premature and low-birth-weight newborns) through delayed cord clamping, which is a simple and strongly effective intervention that can be readily implemented through proper training of healthcare providers (33, 152). It is important to acknowledge that iron supplements are recommended for these target vulnerable groups, regardless of age of introduction of complementary foods.

The persistent reports of insufficient milk supply by mothers as a reason for supplementing breast milk with infant formula can be explained by the lack of adequate lactation management and social support rather than for primary biological reasons that cannot be properly addressed through sound lactation management. Studies have shown that lactation support during the first days and weeks after birth is central to successful establishment of lactation and to minimizing the risk of subsequent lactation difficulties (77, 78). Indeed, SRIM is most likely a result of interference with the normal lactation processes, beginning at birth and often associated with hospital practices (such as unnecessary separation of mother and baby at birth, cesarean delivery without quality breastfeeding support, and in-hospital supplementation policies) and/or cultural norms that encourage pre-lacteal feeds (such as giving newborns water or other ritual liquids) (70, 72, 73, 106, 153). Therefore, successful implementation of the EBF-6 recommendation relies on having a qualified work force and appropriate social protection policies in place to protect, support, and promote breastfeeding since gestation (154).

This Perspective also reaffirms that women with mild to moderate malnutrition, who comprise the majority of undernourished women in LMICs, can produce enough milk volume provided infant demand is not restricted because milk synthesis is dependent more on infant suckling than on maternal nutritional status itself. Breastfeeding is highly protective against infant morbidity and mortality in settings where infectious diseases and undernutrition are common, and therefore is strongly recommended even though the amounts of some micronutrients in mothers’ milk may be suboptimal (155). It is important to reiterate that whereas some of the micronutrients in milk are affected by maternal diet (most of the vitamins, plus iodine and selenium), others are not (most of the minerals, including iron and zinc).

Moving forward, improving maternal nutrition should be a top global public health priority, not only to improve the quality of the milk for infant consumption (especially group 1 nutrients), but also to improve the nutrition and wellbeing of the mother herself.

The child-development literature confirms that there is variability in the age at which infants achieve the necessary developmental milestones to be introduced to complementary foods, and that, on average, this happens at around 6 mo. Parents, other caregivers, and health providers should be taught how to identify the developmental cues that signal readiness for complementary feeding.

All newborns should have their growth, hydration status, and development regularly monitored. In the exceptional circumstances where formula supplementation may be called for, it should be done with careful oversight from a qualified provider, keeping in mind that early breastfeeding problems and corresponding introduction of infant formula are major risk factors for the early termination of both exclusive breastfeeding and any breastfeeding (74, 91, 156–160).

Further research is needed to continue developing standards for human milk production, consumption, and composition in diverse settings and at different stages of lactation (77, 99). It is possible that within a population some women are more predisposed (genetically or otherwise) to experience insufficient milk when subjected to risk factors for a suboptimal milk volume. Research is needed to identify at-risk women so they can be offered additional breastfeeding support and close monitoring of their infants’ growth and development.

A limitation of our Perspective is that we did not address the breastfeeding needs and outcomes among women in extreme circumstances such as starvation, or experiencing chronic diseases. Because that scope falls beyond the public health recommendation for EBF-6 and requires a personalized clinical approach, it is important that future reviews focus on those topics.

In conclusion, the 2001 WHO EBF-6 recommendation was issued as a public health recommendation, which acknowledged that tailoring to individual infants’ circumstances would be required (3), and that these needs should be determined by regular growth and developmental monitoring. Existing, evidence-based responsive feeding guidelines provide recommendations on how best to determine when an infant is ready to be introduced to complementary foods based on her/his developmental stage (139). We suggest that infant and young feeding guidelines make clear that complementary foods should be introduced at around age 6 mo, taking infant developmental readiness into account, but that no other modifications be made to the EBF-6 public health recommendation based on the concerns reviewed in this Perspective paper. Research will help refine clinical guidelines on infant feeding in vulnerable populations in the future.

## Notes

Perspective articles allow authors to take a position on a topic of current major importance or controversy in the field of nutrition. As such, these articles could include statements based on author opinions or point of view. Opinions expressed in Perspective articles are those of the author and are not attributable to the funder(s) or the sponsor(s) or the publisher, Editor, or Editorial Board of *Advances in Nutrition*. Individuals with different positions on the topic of a Perspective are invited to submit their comments in the form of a Perspectives article or in a Letter to the Editor.

Supported by the Bill & Melinda Gates Foundation.

Author disclosures: RP-E, GSB, and SS-P, no conflicts of interest. EP works for the Bill & Melinda Gates Foundation, which funded this Perspective.

Abbreviations used: DOL, delayed onset of lactation; EAT, Enquiring about Tolerance; EBF, exclusive breastfeeding; EBF-6, exclusive breastfeeding for 6 mo; HMO, human milk oligosaccharide; LBW, low birth weight; LMIC, low-income/middle-income country; RCT, randomized controlled trial; SRIM, self-reported insufficient milk.
